# Prediction of the Factors Influencing the Shengjing Classification of Portal Vein Thrombosis after Splenectomy for Portal Hypertension in Cirrhosis: A Single-Center Retrospective Case-Control Study

**DOI:** 10.1155/2020/2396710

**Published:** 2020-09-07

**Authors:** Zimin Song, Hanxin Feng, Chunbo Yang, Chaoliu Dai

**Affiliations:** Department of General Surgery, Shengjing Hospital of China Medical University, No. 36, Sanhao Street, Heping District, Shenyang, 110004 Liaoning Province, China

## Abstract

**Objective:**

To compare the survival time of patients with portal vein thrombosis after splenectomy for portal hypertension in cirrhosis and explore the influencing factors of the Shengjing classification.

**Methods:**

Clinical data of 108 patients with portal vein thrombosis after splenectomy in the department of general surgery of our hospital from November 2011 to December 2018 were selected, and a retrospective analysis was performed.

**Results:**

Among 108 patients with postoperative PVST formation, 9 had type Ia, 32 type Ib, 39 type IIa, 20 type IIb, 5 type IIIa, 3 type IIIb, and 0 type IV. Survival analysis showed that the difference in survival time distribution among the Shengjing typing groups was statistically significant (*P* < 0.05). The higher the classification level, the shorter the survival time and the higher the risk of death. The results of a single-factor analysis showed that there were statistically significant differences in the PVST Shengjing typing groups between the preoperative group with or without hepatitis, preoperative d-dimer level, and postoperative day 14 fibrinogen (FIB) level (*P* < 0.05). Multivariate logistic regression analysis showed that the OR value of higher PVST Shengjing typing in patients with hepatitis was 4.634 times higher than that in patients without hepatitis (95% CI: 1.593-13.478, *χ*^2^ = 7.922, *P* = 0.005 < 0.05). Preoperative d-dimer volume increased by 1 *μ*g/L; the OR value of higher grade PVST Shengjing typing was 1.001 times higher (95% CI: 1.000-1.002) than that of lower grade PVST Shengjing typing (*χ*^2^ = 8.369, *P* = 0.004 < 0.05).

**Conclusions:**

The survival time of patients with portal vein system thrombosis after splenectomy was significantly different among Shengjing typing groups, and the higher the classification level, the shorter the survival time and the higher the risk of death. In patients with portal hypertension in cirrhosis and PVST formation after splenectomy, if the preoperative d-dimer level is high or accompanied by hepatitis virus, the formation of PVST will involve a wide range, the disease is more serious, and the prognosis is also poor, so corresponding preventive measures should be taken to avoid the aggravation of PVST.

## 1. Introduction

Portal vein system thrombosis (PVST) formation is easy to occur in patients with portal hypertension in cirrhosis after splenectomy. It can lead to obstruction of blood flow reflux in the portal vein system, further aggravating liver function injury; severe PVST can lead to liver failure, rupture of the esophageal and gastric fundus, refractory ascites, and even life-threatening intestinal necrosis. However, due to its complex risk factors, clinical symptoms are not obvious, and diagnosis is difficult, so it is easy to lead to misdiagnosis and missed diagnosis.

**Figure 1:**
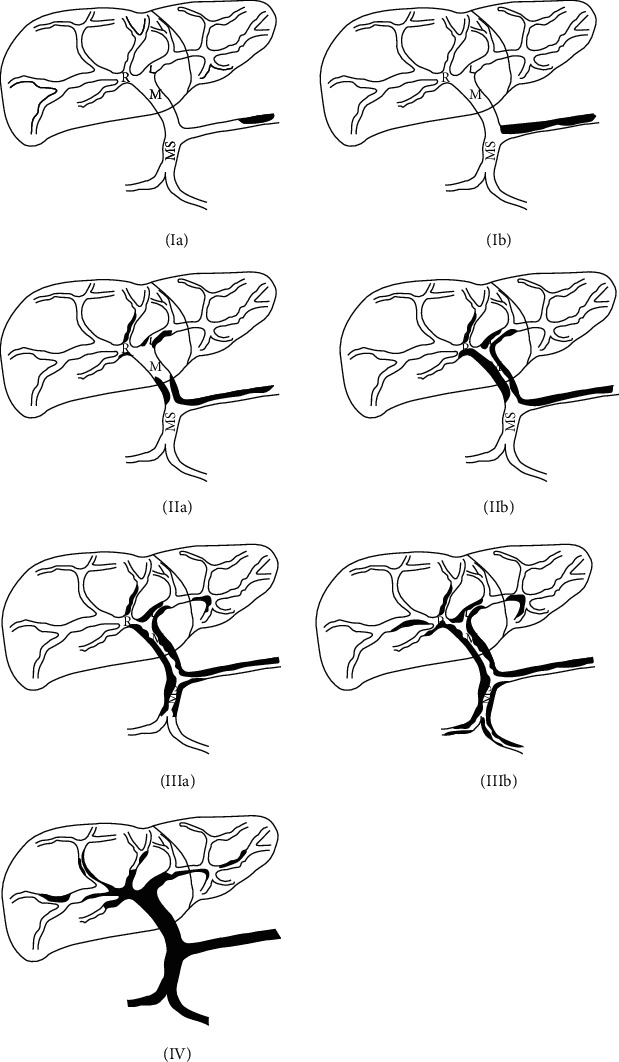
Schematic diagram of PVST Shengjing classification after splenectomy for portal hypertension of cirrhosis.

Any thrombosis in the portal system of the main portal vein and its left and right branches, splenic vein, and superior mesenteric vein is defined as portal vein system thrombosis (PVST). PVST is the most serious and common complication after splenectomy for portal hypertension in cirrhosis; it is usually diagnosed by postoperative abdominal ultrasound, CT (computed tomography), or MRI (magnetic resonance imaging), presenting with a portal vein system filling defect. According to literature reports, the natural formation rate of portal vein thrombosis in patients with portal hypertension in cirrhosis is about 0.6%~12.1% [1]. The incidence of PVST after splenectomy was about 0.5%~57% [2, 3], and some domestic scholars reported that the incidence was as high as 91.06% [4]. Classification of PVST is particularly important for its diagnosis and treatment. Currently, the Yerdel bispecking method is widely recognized. However, all of them were PVST classification of nonsurgical patients, while the Shengjing classification proposed by our department (Table 1, Figure 1) was specific to PVST classification after splenectomy and further refined compared with other classifications [5]. It is convenient to accurately judge the severity of PVST and choose reasonable treatment in the follow-up. In this study, clinical data of 108 patients with postoperative PVST formation were retrospectively analyzed to explore the influencing factors of PVST Shengjing typing so as to improve the diagnosis and treatment of portal vein thrombosis.

## 2. Patients and Methods

### 2.1. Patients

This retrospective study collected clinical data of 108 patients with portal hypertension due to cirrhosis who were admitted to the department of general surgery of our hospital from November 2011 to December 2018 and had postoperative PVST formation; all patients underwent laparotomy, including 9 who underwent splenectomy alone and 99 who underwent splenectomy plus gastric fundus and pericardial devascularization. The age ranged from 14 to 66 years, with an average age of 48.44 ± 10.68 years. As regards liver function Child grade, there are 87 cases of grade A and 21 cases of grade B. All patients were diagnosed with portal hypertension, including 73 cases of hepatitis B cirrhosis, 12 cases of hepatitis C cirrhosis, 13 cases of cryptogenic cirrhosis, 7 cases of alcoholic cirrhosis, and 3 cases of biliary cirrhosis. None of the patients received preventive anticoagulant therapy after surgery, and standardized systemic anticoagulant therapy was immediately adopted after the presence of PVST was confirmed by imaging, such as urokinase thrombolytic therapy and subcutaneous injection of Low Molecular Weight Heparin (LMWH) or oral warfarin to prevent recurrence of PVST.

The collected clinical data were divided into preoperative data, intraoperative data, and postoperative data. Preoperative data included age, gender, history of diabetes, etiology of cirrhosis, Child grade of liver function, preoperative portal and splenic vein diameter, preoperative platelet level, preoperative coagulation index, and preoperative liver function index. The intraoperative data included whether or not the splenectomy was anatomic, the operation method, the operation time, the splenic index, the intraoperative blood loss, and the intraoperative blood transfusion. Postoperative data included the postoperative portal splenic vein diameter; postoperative drainage volume; postoperative platelet level at the 1st, 7th, or 14th day; and postoperative coagulation index on the 1st, 7th, or 14th day.

### 2.2. Inclusion Criteria

The inclusion criteria were as follows: (1) liver cirrhosis was confirmed by CT, MRI, or ultrasound; (2) gastroscopy or barium meal confirmed that there were varicose veins in different degrees around the stomach and esophagus; and (3) hypersplenism is present.

### 2.3. Exclusion Criteria

Individuals were excluded based on the following criteria: (1) malignant tumors of the liver, pancreas, and gastrointestinal tract; (2) hematogenous diseases; (3) preoperative imaging examination has found thrombus in the portal vein system; (4) congenital portal venous malformation; (5) incomplete clinical data; and (6) previous history of abdominal surgery.

The study was registered on the World Health Organization International Clinical Trials Registry Platform (ChiCTR2000029274) and was conducted in conformity with the Declaration of Helsinki [18].

### 2.4. Detection and Diagnosis of PVST

All 108 patients underwent enhanced spiral CT, MRI, or color Doppler ultrasound before and after surgery. All patients had no PVST in the preoperative imaging examination. Imaging reexamination was conducted from day 2 to day 14 after surgery. The diagnosis of PVST was made when the filling defect of the portal vein system was found on the imaging examination. According to the Shengjing classification criteria of thrombus, PVST was divided into four types: Shengjing types I, II, III, and IV. All clinical and radiographic examinations were measured independently by three examiners (Z.M.S., H.X.F., and C.B.Y.) who were unaware of the status. These examiners had been trained using the same standard and could achieve consistent examination results. The *k* coefficients, or intraclass correlation coefficients between examiners, ranged from 0.83 to 0.92 for the preoperative portal and splenic vein diameter and from 0.77 to 0.85 for the postoperative portal splenic vein diameter.

### 2.5. Statistical Analyses

SPSS 20.0 software was used. The measurement data were expressed as *x* ± *s*. One-way ANOVA was used. If the measurement data are not normally distributed or have uneven variances, the Kruskal-Wallis *H* test is performed. If the rate of counting data is expressed, use the*χ*^2^test or Fisher's exact test. Multivariate logistic regression analysis was used for multivariate analysis. Mean = 0.05 and *P* < 0.05 indicated that the difference was statistically significant. The survival curves of different subgroups were plotted by the Kaplan-Meier method, and the log-rank method was used for the statistical test. The test level was equal to 0.05, and the COX regression model was used for multifactor analysis.

## 3. Results

### 3.1. The Incidence of PVST and Shengjing Classification

In this study, a total of 278 patients were collected, of which 108 developed thrombus in the portal vein system postoperatively, and the thrombus formation rate was 38.8%. Among the 108 patients with postoperative PVST formation, 9 had type Ia, 32 type Ib, 39 type IIa, 20 type IIb, 5 type IIIa, 3 type IIIb, and 0 type IV. The 108 patients in this study had no other complications requiring special treatment except conventional postoperative anticoagulation therapy.

### 3.2. Survival Analysis

**Figure 2:**
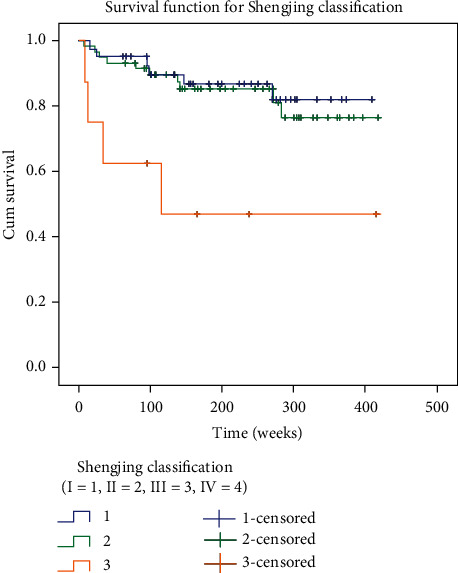
Survival curve of the Shengjing classification Kaplan-Meier method.

The Kaplan-Meier method was used to compare the quality of life of patients with Shengjing classification type I, type II, and type III. The deletion rates of type I, type II, and type III patients were 85.4%, 81.7%, and 57.1%, respectively; the distribution was similar in each group. The survival curves of patients with Shengjing types I, II, and III are shown in Figure 2. It can be generally seen that patients with type I have a longer survival time than patients with type II and type III, and patients with type II have a longer survival time than patients with type III. The results of the log-rank test showed a statistically significant difference in the distribution of survival time among the three groups (*χ*^2^ = 9.529, *P* = 0.009 < 0.05). Pairwise comparison showed a statistically significant difference in the distribution of survival time between the type I and type III patients (*χ*^2^ = 5.405, *P* = 0.02 < 0.05); the difference in the distribution of survival time between the type II and type III patients was also statistically significant (*χ*^2^ = 4.087, *P* = 0.043 < 0.05). However, there was no significant difference in the distribution of survival time between the type I and type II patients (*χ*^2^ = 0.281, *P* = 0.596 > 0.05). To exclude the influence of confounding factors on survival analysis, age and gender were incorporated into the COX proportional risk model for analysis. As shown in Table 2, there were significant differences in the distribution of survival time among groups after considering age and gender; the score statistics was *χ*^2^ = 9.529, *P* = 0.009 < 0.05, indicating that the model test was statistically significant. Compared with type I and type II, *P* = 0.01 < 0.05, HR (relative risk) = 0.187, and the 95% confidence interval of HR is 0.052-0.669, indicating that the risk of death of patients with type I is 0.189 times that of patients with type II. Compared with type II and type III, *P* = 0.012 < 0.05, HR (relative risk) = 0.225, and the 95% confidence interval of HR is 0.070-0.722, indicating that the risk of death of type II patients is 0.225 times that of type III patients.

### 3.3. One-Way Analysis of Variance and Chi-Squared Test

The results of one-way ANOVA and the chi-squared test of preoperative factors that may affect the Shengjing classification are shown in Table 3. The analysis results showed that the preoperative presence or absence of hepatitis virus was a factor with a statistically significant difference among the Shengjing classification groups (*F* = 9.571, *P* = 0.011 < 0.05); among them, the difference between the Shengjing type III and type I groups whether the patients had hepatitis virus or not was statistically significant (*P* < 0.05), while the difference between the type II and type I groups and between the type III and type II groups was not statistically significant (*P* > 0.05). The analysis results also showed that the preoperative d-dimer level of the Shengjing classification typing I group was 266.37 ± 434.845 *μ*g/L, the preoperative level of d-dimer in the type II group was 364.24 ± 493.458 *μ*g/L, and the preoperative level of d-dimer in the type III group was 1013.88 ± 1163.257 *μ*g/L. The difference in the preoperative d-dimer level among the three groups of type I, type II, and type III was statistically significant (*F* = 3.660, *P* = 0.029 < 0.05), and pairwise comparisons between groups show that there was a statistically significant difference in the preoperative d-dimer level between the type III and type I groups (*P* < 0.05); however, there was no significant difference in the preoperative d-dimer level between the type III and type II groups or between the type II and type I groups (*P* > 0.05). The results of one-way ANOVA and the chi-squared test of intraoperative factors that may affect the Shengjing classification are shown in Table 3; the analysis results showed that there were no statistically significant differences in the intraoperative influencing factors among the groups (*P* < 0.05). The results of one-way ANOVA of postoperative factors that may affect the Shengjing classification are shown in Table 3; the analysis results showed that the FIB (fibrinogen) level on the 14th day after the operation was 2.39 ± 0.647 g/L in the type I group, 2.75 ± 0.853 g/L in the type II group, and 2.29 ± 0.98 g/L in the type III group, and the FIB level on the 14th day after surgery was significantly different among the three groups of type I, type II, and type III (*F* = 3.203, *P* = 0.045 < 0.05). Pairwise comparisons between groups show that the difference of the FIB level on the 14th day after surgery between the type III and type I groups was statistically significant (*P* < 0.05); there was no significant difference in the FIB level between the type II and type I groups or between the type III and type II groups on the 14th day after surgery (*P* > 0.05).

### 3.4. Ordered Multiple Classification Logistic Regression Analysis

Factors that were statistically significant using one-way ANOVA and the chi-squared test were introduced into the ordered multiclassification logistic regression model, ordered multiclassification logistic regression analysis was performed, and the analysis results are shown in Table 4. In the parallel line test (*χ*^2^ = 4.088, *P* = 0.252), the parallelism hypothesis was established, and the regression equations were parallel to each other. The deviance goodness of fit test showed that the model fitted well (*χ*^2^ = 173.051, *P* = 0.959), but there were (66.7%) cells with 0 frequency. The goodness of fit test showed that this model was superior to the model with only constant terms (*χ*^2^ = 19.356, *P* < 0.001). The OR value of forming higher grade typing in patients with hepatitis was 4.634 times higher than that in patients without hepatitis (95% CI: 1.593-13.478, *χ*^2^ = 7.922, *P* = 0.005 < 0.05). For each increase of 1 *μ*g/L in the preoperative d-dimer volume, the OR value of the higher grade PVST Shengjing classification was 1.001 times higher than that of the lower grade PVST Shengjing classification (95% CI: 1.000-1.002, *χ*^2^ = 8.369, *P* = 0.004 < 0.05).

## 4. Discussion

Scholars at home and abroad have long studied the classification of portal vein thrombosis. Since 1991, Stieber et al. [6] proposed the Stieber classification of the portal vein system; at least 10 classification methods have been proposed [7]. However, most of these methods only focus on anatomical analysis and lack of functional analysis and do not put forward the corresponding prevention or treatment. Moreover, all previous portal thrombosis classification methods were only focused on patients with nonsurgical cirrhosis; however, the portal vein thrombosis classification proposed by our department is mainly targeted at patients with portal hypertension after splenectomy; based on the analysis of the advantages and disadvantages of previous thrombus classification methods, the Shengjing classification is further optimized compared with previous classifications (Figure 1). It is more convenient for the prevention, diagnosis, and treatment of postoperative portal vein thrombosis, which is beneficial to improve the diagnosis and treatment level of portal vein thrombosis after splenectomy for portal hypertension in cirrhosis.

It is well known that the wider the involvement of the portal vein system after thrombosis, the worse the patient's prognosis. If PVST only involves the splenic vein, it has no significant effect on mesenteric venous reflux. With the pathogenesis of thrombosis, the prognosis of patients is better. However, if the main portal vein or even the superior mesenteric vein is involved, venous reflux will be affected, and intestinal ischemia and infarction may be potentially fatal [8], so the prognosis is poor. Shengjing classification was divided into several grades according to the extent of thrombus involvement; the higher the level, the wider the scope of involvement. This study also showed that there were significant differences in survival time among different types of the Shengjing classification; the higher the grade, the greater the risk of death. This also proves the scientific nature and rationality of the Shengjing classification. For patients with portal hypertension following splenectomy, the severity of thrombosis can be evaluated according to the Shengjing classification, and then targeted treatment methods can be selected to reduce the risk of further aggravation of thrombosis, which plays an important role in the diagnosis, treatment, and prognosis of thrombosis in the portal vein system after splenectomy.

The rise of d-dimer has been considered to be one of the main factors for thrombosis of the portal vein system after splenectomy. Some scholars reported that the increased d-dimer level after splenectomy was an independent risk factor for the formation of PVST [9]. The increase of d-dimer after surgery indicates hypercoagulability and fibrinolytic hyperactivity of the body. The body is in a state of high coagulation, which is more likely to form PVST. This study also showed that the difference of the preoperative d-dimer level between different types of the Shengjing classification groups was statistically significant. Specifically, there was a statistically significant difference in the preoperative d-dimer level between the type III and type I groups (*P* < 0.05); the results showed that the preoperative d-dimer level had a predictive effect on postoperative thrombus involvement. Further analysis showed that for each increase of 1 *μ*g/L in preoperative d-dimer volume, the probability of patients forming a higher grade PVST Shengjing classification was 1.001 times higher than that of a lower grade PVST Shengjing classification; that is, the higher the preoperative d-dimer, the more extensive the portal vein system involved by PVST formed after the operation and the more serious the disease is.

According to studies, patients with chronic hepatitis may have an increased risk of portal vein thrombosis [10, 11]. Some scholars believe that the formation of PVST may be related to chronic viral infection-mediated inflammation and hemostatic disorders [12]. Studies have also shown that patients with chronic viral hepatitis can produce antiphospholipid antibodies, which are related to thrombosis of the portal vein system [13, 14]. This study showed that there was a statistically significant difference among the Shengjing classification groups in whether the patients were accompanied with hepatitis virus before surgery (*P* < 0.05). Pairwise comparison indicated that whether the patients had hepatitis virus or not, the difference between the Shengjing analysis type III and I groups was statistically significant (*P* < 0.05).

Further analysis showed that patients with preoperative hepatitis were 4.231 times more likely to form a higher PVST Shengjing classification than patients without preoperative hepatitis. Patients with hepatitis virus had a wider range of PVST accumulations after surgery, suggesting a more severe postoperative outcome and a poorer prognosis.

It has been reported that increased risk of venous thrombosis is associated with increased fibrinogen concentration [15]. Some scholars also believe that changes in FIB structure and function in patients with cirrhosis, including changes in carbohydrate and an increase in carbonyl content, lead to abnormal fibrin network structure and stability and increase the risk of postoperative portal vein thrombosis [16, 17]. The univariate analysis of this study showed that the FIB level on the 14th day after surgery was statistically significant in PVST Shengjing classification (*P* < 0.05). Specific pairwise comparison showed that the difference in the FIB level on the 14th day after surgery between the type III and type I groups was statistically significant (*P* < 0.05). The FIB level on the 14th day in the type I group was higher than that in the type III group, and the multivariate logistic regression analysis found that the FIB level on the 14th day after the operation showed no statistically significant difference among different groups. This indicates that compared with the FIB level on the 14th day, it is possible that the function change of FIB is the main factor influencing the formation of postoperative PVST.

In conclusion, the Shengjing classification plays an important role in the diagnosis, treatment, and prognosis of portal vein thrombosis. If the d-dimer is high before surgery or accompanied by hepatitis virus, the PVST formed after surgery will involve a wider range, the disease is more serious, and the prognosis is also poor. Therefore, corresponding preventive measures should be taken to avoid the aggravation of PVST.

This study mainly explored the factors influencing the Shengjing classification of portal vein thrombosis after splenectomy, and the differences in prognosis and survival among the different groups were analyzed. The explored new criteria for the diagnosis, treatment, and prevention of portal vein thrombosis after splenectomy improved the level of diagnosis and treatment of the disease. Of course, this study also has its shortcomings. First of all, due to the limitations of the conditions, the number of samples that meet the research conditions is not enough, resulting in the absence of type IV patients. Furthermore, this study is a single-center study, and its conclusions may be influenced by geography, environment, or customs, so it is not well represented. Therefore, in view of the above deficiencies, the next step is to expand the scope of our study, adopt a multicenter study, and include eligible patients with type IV in the study so as to make the research conclusions more reasonable and more representative.

## Figures and Tables

**Table 1:** Shengjing classification of portal vein thrombosis after splenectomy.

Classification	PS-PVST	Therapies					
		Observe	Remove gathered	Anticoagulation	Thrombolysis	Intervention	Surgery
I	The thrombus is confined to the splenic vein						
Ia	The thrombus is less than 1/2 distal to the splenic vein	✓	✓				
Ib	The thrombus extends to 1/2 of the proximal splenic vein	✓	✓	✓			
II	The thrombus extends to the portal vein but is not completely blocked						
IIa	The range is less than 1/2 of the main portal vein and/or thrombosis of the left or right branch of the portal vein	✓	✓	✓	✓		
IIb	The range exceeds 1/2 of the main portal vein, and/or the left or right branch of the portal vein is thrombotic, but not yet completely blocked	✓	✓	✓	✓		
III	The thrombus involved the superior mesenteric vein but was not completely obstructed						
IIIa	Thrombosis involves the main superior mesenteric vein	✓	✓	✓	✓	✓	
IIIb	The thrombus extends to the superior mesenteric vein branch	✓	✓	✓	✓	✓	✓
IV	The portal vein system (main or left or right branches of the portal vein or superior mesenteric vein) is completely blocked	✓	✓	✓	✓	✓	✓

**Table 2:** COX regression analysis results of the Shengjing classification.

Variables	*β*	SE	Wald	*P*	HR	HR 95% CI	
						Lower limit	Upper limit
Shengjing classification I/II	-1.675	0.649	6.656	0.010^a^	0.187	0.052	0.669
Shengjing classification II/III	-1.492	0.595	6.293	0.012^a^	0.225	0.070	0.722

^a^ *P* < 0.05 indicates that the difference in mortality risk between the Shengjing classification groups was statistically significant. HR: relative risk.

**Table 3:** One-way ANOVA and chi-squared test of influencing factors.

Variables	Type I (*n* = 41)	Type II (*n* = 59)	Type III (*n* = 8)	*P* values
Hepatitis				0.006^a^
Yes	38 (92.7)	44 (74.6)	4 (50.0)^#^	
No	3 (7.3)	15 (25.4)	4 (50.0)	
Preoperative DD (*μ*g/L)	266.37 ± 434.845	364.24 ± 493.458	1013.88 ± 1163.257^#^	0.029^a^
FIB (g/L)on the 14th day after surgery	2.390 ± 0.647	2.750 ± 0.853^∗^	2.290 ± 0.980	0.045^a^

^#^ *P* < 0.05, the difference of influencing factors between type III and type I was statistically significant. ^∗^*P* < 0.05, the difference of influencing factors between type II and type I was statistically significant. ^a^*P* < 0.05 means one-way ANOVA has statistical significance.

**Table 4:** Ordered multiple classification logistic regression analysis.

Independent variables	*β*	SE	Wald	*P*	OR	OR 95% CI	
						Lower limit	Upper limit
Hepatitis (yes/no)	1.533	0.5448	7.922	0.005^a^	4.634	1.593	13.478
Preoperative DD (*μ*g/L)	0.001	0.0004	8.369	0.004^a^	1.001	1.000	1.002
FIB (g/L) on the 14th day after surgery	0.344	0.2484	1.914	0.166	1.41	0.867	2.294

^a^ *P* < 0.05 indicates that the influencing factor had statistical significance in the logistic regression analysis of ordered multiple classification.

## Data Availability

The data used to support the findings of this study are included in the article.
